# Movement-Related Sensorimotor High-Gamma Activity Mainly Represents Somatosensory Feedback

**DOI:** 10.3389/fnins.2017.00408

**Published:** 2017-07-14

**Authors:** Seokyun Ryun, June S. Kim, Eunjeong Jeon, Chun K. Chung

**Affiliations:** ^1^Interdisciplinary Program in Neuroscience, Seoul National University College of Natural Sciences Seoul, South Korea; ^2^Department of Brain and Cognitive Sciences, Seoul National University College of Natural Sciences Seoul, South Korea; ^3^Department of Neurosurgery, Seoul National University College of Medicine Seoul, South Korea

**Keywords:** voluntary movement, primary somatosensory cortex, primary motor cortex, high-gamma activity, somatosensory feedback, electrocorticography (ECoG), brain-machine interface (BMI)

## Abstract

Somatosensation plays pivotal roles in the everyday motor control of humans. During active movement, there exists a prominent high-gamma (HG >50 Hz) power increase in the primary somatosensory cortex (S1), and this provides an important feature in relation to the decoding of movement in a brain-machine interface (BMI). However, one concern of BMI researchers is the inflation of the decoding performance due to the activation of somatosensory feedback, which is not elicited in patients who have lost their sensorimotor function. In fact, it is unclear as to how much the HG component activated in S1 contributes to the overall sensorimotor HG power during voluntary movement. With regard to other functional roles of HG in S1, recent findings have reported that these HG power levels increase before the onset of actual movement, which implies neural activation for top-down movement preparation or sensorimotor interaction, i.e., an efference copy. These results are promising for BMI applications but remain inconclusive. Here, we found using electrocorticography (ECoG) from eight patients that HG activation in S1 is stronger and more informative than it is in the primary motor cortex (M1) regardless of the type of movement. We also demonstrate by means of electromyography (EMG) that the onset timing of the HG power in S1 is later (49 ms) than that of the actual movement. Interestingly, we show that the HG power fluctuations in S1 are closely related to subtle muscle contractions, even during the pre-movement period. These results suggest the following: (1) movement-related HG activity in S1 strongly affects the overall sensorimotor HG power, and (2) HG activity in S1 during voluntary movement mainly represents cortical neural processing for somatosensory feedback.

## Introduction

In our everyday lives, somatosensation is always induced during any type of active movement. Without this, our ability to control our movement may decrease dramatically (Rothwell et al., 1982; Sanes et al., 1984; Jenmalm and Johansson, 1997). For example, we would be unable to eat comfortably, take something out of a bag, or grasp something with appropriate force. To perform movements dexterously, simultaneous somatosensory feedback and complex sensorimotor interaction are required. In our brain, the primary somatosensory cortex (S1) plays crucial roles in such proprioceptive and tactile somatosensory feedback caused by movement and sensorimotor interaction, such as, an efference copy, to prepare for movement (Christensen et al., 2007). In addition, S1 probably modulates the somatosensory input such as, proprioceptive feedback by sending an efferent signal to the dorsal horn, which does not receive input from the primary motor cortex (M1), through the descending corticospinal tract (Armand et al., 1997; Lemon, 2008).

During proprioceptive/tactile feedback, somatosensory signals elicited by various mechanoreceptors in our body are transmitted to the brain within the order of dozens of milliseconds. In macroscopic electrophysiological recordings, these types of information are often represented by event-related potentials (ERPs) or event-related de-synchronization (ERD) in S1 (Wiest and Nicolelis, 2003; Lim et al., 2012). Further, many invasive studies, such as, electrocorticography (ECoG) and microelectrode recordings, have indicated that there are dominant high-gamma (>50 Hz) power changes in the sensorimotor area during not only passive somatosensory stimulation but also active movement (Miller et al., 2007; Ray et al., 2008a; Avanzini et al., 2016). These activities may represent movement-related somatosensory feedback (Chestek et al., 2013). Although their exact mechanisms remain to be elucidated, previous studies have suggested that somatosensory high-gamma (HG) activities represent the intensity of sensory stimulation and are related to increases in the neuronal firing rate (Ray et al., 2008b; Zhang et al., 2012; Rossiter et al., 2013).

HG activities have also been observed in the M1 during active movement (Crone et al., 1998; Cheyne et al., 2008). Owing to their robustness, recent ECoG-based brain-machine interface (BMI) studies using active movement data have regarded the signals from sensorimotor HG activity as important features. However, one concern in these BMI studies is the inflation of the decoding performance due to the activity from somatosensory feedback (Chestek et al., 2013; Bleichner et al., 2016), as the decoding performance by the S1 signal alone is sufficient compared to that by the M1 signal. Furthermore, it is unclear as to how much these somatosensory activities contribute to the overall level of sensorimotor HG power. Practically and specifically, this issue is important because if these activities mainly come from the somatosensory area and represent somatosensory feedback, HG-based BMI systems may not be sufficient when attempting to make accurate estimations of movements given that the major candidates of such BMI users would be people who have lost some sensorimotor function. To the best of our knowledge, no investigations thus far have quantified the influence of HG activity in S1 compared to that in M1 during voluntary movement with a sufficient number of ECoG patients.

Emerging evidence related to the functional roles of S1 indicates that S1 activities are associated with movement prediction and preparation by top-down mechanisms from the premotor and primary motor cortices to S1 (Christensen et al., 2007; Adams et al., 2013). Although the relevance of the HG activity with regard to these functional roles is somewhat controversial, recent studies have proposed that there exists a significant increase in the S1 HG power level before a cued movement, which represents neuronal information decoupled from somatosensory feedback, or an efference copy (Sun et al., 2015; Hotson et al., 2016; Branco et al., 2017). However, these promising results have not been confirmed in a fully voluntary movement case and with electromyography (EMG), which can detect exact movement onset times from physiological fluctuations, thereby avoiding certain undesirable circumstances such as, isometric muscle contractions.

To address these questions, we recorded human ECoG data in several areas including the primary somatosensory and motor cortices, during cued or voluntary movement from a relatively large number of patients. We investigated whether the overall sensorimotor HG power levels during active movement mainly come from S1 or M1. We also tested how these power changes from a somatosensory area affect movement classification performance outcomes. Finally, we investigated the timing of the movement-related S1 HG activity with EMG signals to confirm whether the activity mainly represents somatosensory feedback or whether it also contains significant information about pre-movement neural interactions.

## Materials and methods

### Subjects

Eleven patients (5 female, aged 21–36 years) with intractable epilepsy were included in this study. Patients underwent implantation of subdural electrode grids/strips (Ad-tech Medical Instrument, Racine, WI, USA) to localize the seizure focus areas. The electrodes used had a diameter of 4 mm with an inter-electrode distance of 10 mm. The electrodes in each case covered sensorimotor-related brain areas, including both the primary motor and somatosensory cortices. Preoperative magnetic resonance (MR) and postoperative computed tomography (CT) images were obtained from each subject. MR-CT image co-registration was performed to localize ECoG electrodes, and these electrodes were overlapped with individual 3-D brain structures using the CURRY software (version 7.0, Compumedics Neuroscan, Charlotte, NC, USA). Electrode locations were determined by careful visual inspections based on MR-CT co-registration images and 3-D brain structures. If some electrodes were located on the central sulcus, the closest gyrus was chosen. All patients (except Subject 8) reported sensorimotor experiences related to the hand/finger and arm when the electrical stimuli were delivered to the electrodes, which are located in the sensorimotor area during direct cortical stimulation for clinical purpose (Supplementary Figure 1). All experimental procedures were approved by the Institutional Review Board of Seoul National University Hospital (H-0912-067-304). All patients signed informed consent forms before their participation. See Table 1 for patient details.

**Table 1:** Clinical profiles.

**Subject**	**Experiment**	**Electrodes location/number**	**Diagnosis**
1	HG, EF	L hemisphere/72	FLE
2	HG, EF	R hemisphere/52	TLE
3	HG, EF	L hemisphere/48	TLE
4	HG, EF	R hemisphere/68	OLE
5	HG, EF	L hemisphere/82	PLE
6	HG, EF	L hemisphere/64	TLE
7	HG, EF	R hemisphere/64	OLE
8	HG, EF, V	R hemisphere/84	TLE
9	Reaching	L hemisphere/50	FLE
10	Reaching	Bilateral/68	FLE
11	Reaching	R hemisphere/56	TLE

*F, female; M, male; HG, hand grasping; EF, elbow flexion; V, vibrotactile; L, left; R, right; FLE, frontal lobe epilepsy; TLE, temporal lobe epilepsy; OLE, occipital lobe epilepsy; PLE, parietal lobe epilepsy*.

### Tasks

Eight patients performed self-paced, voluntary hand grasping, and elbow flexion motions, as described in earlier work (Ryun et al., 2014), contralateral to the implantation site. We instructed the patients to move their hands/arms at approximate intervals of 5–10 s, but not to count the number of seconds during the resting period (mean interval across all subjects and trials: 10.43 ± 5.09 s; mean ± standard deviation). At the initial condition, patients placed their hands/arms comfortably on the patient table with their palms upward. For hand grasping motion, patients were asked to perform hand grasping motion with no object, and then release their hands after that motion (1–2 s). For elbow flexion, we instructed the patients to flex their arms until the limit of the joint, and extend them with minimal force. To obtain good task performances, we instructed the patients to practice these tasks for 2 min. Each session consisted of 17–51 trials, and patients performed two to four sessions per movement type. The mean durations of each movement types were 2.84 ± 1.01 and 3.63 ± 1.06 s for hand grasping and elbow flexion, respectively. To detect on/offset of the movements, we recorded EMG signals from the opponens pollicis for hand grasping and from the biceps brachii for elbow flexion motions. All experiments were recorded on video to monitor the task performance process. The total number of sessions across all subjects was 46, but we excluded three sessions due to the extremely low signal-to-noise ratio of the EMG. One patient also participated in a vibrotactile stimulation experiment. Stimuli were delivered to the index fingertip contralateral to the implantation site. We stimulated six different vibrotactile frequencies (5, 20, 35, 100, 250, and 400 Hz; 50 trials per condition) with a stimulus duration of 1 s and with an inter-stimulus interval of 2.5, 3, or 3.5 s.

Three patients performed a center-out, three-dimensional reaching movement task. The details of this task procedure are illustrated in the literature (Yeom et al., 2013). Briefly, patients started to move their arms contralateral to the implantation sites after visual cues which indicated the target of movement. The target was pseudo-randomly presented in each of four directions, and one session consisted of 30 trials for each direction. Movement onsets and trajectories were detected using a three-axis accelerometer (KXM52, Kionix, NY, USA). The sensors were attached to the index finger to estimate the end point of movement, and the corresponding signals were recorded simultaneously with ECoG signals. No EMG recording was performed in this experiment.

### Data acquisition and preprocessing

ECoG signals were recorded using the 128-channel Natus Telefactor (Telefactor Beehive Horizon with an AURA® LTM 64 & 128-channel amplifier system, Natus Neurology, West Warwick, RI, USA) or Neuroscan (Neuroscan, Charlotte, NC, USA) amplifier systems. The ECoG electrodes, which show abnormal signals due to technical problems and epileptiform activities, were excluded from any further analysis. Signals were digitized at sampling frequencies of 200 (Subject 1), 400 (Subjects 2 and 3), 1,000 (Subjects 6–11), and 1,600 Hz (Subjects 4 and 5). Before the analog-to-digital conversion, we undertook analog anti-alias filtering ranging from 0.1 to 80, 150, 200, and 400 Hz for sampling rates of 200, 400, 1,000, and 1,600 Hz, respectively.

For the hand grasping and elbow flexion study, we initially determined the on/offset of each movement using both an automated detection algorithm based on a threshold method and careful visual inspections. EMG data were band-pass filtered from 20 to 70 Hz. We then applied the Hilbert transform to the filtered data and took the absolute value from the analytic signal to calculate the envelope signal. With the detection algorithm, the on/offset points were roughly selected, and epoching was performed with a window of 2.5 s before onset to 2.5 s after offset. After epoching, we determined the exact on/offset points by means of visual inspection. Time points between resting and bursting EMG time traces were chosen for the movement on/offsets. These data were normalized by the resting period signals (−2 to −1 s of movement onset). Throughout the detection procedure, trials which showed low signal-to-noise ratios due to poor contact or other technical problems were excluded from any further analysis (97 of 1,342 trials, 7.23%; 1,245 valid trials). See Table 2 for valid behavior information.

**Table 2:** Behavior information.

**Subject**	**Hand grasping**			**Elbow flexion**		
	**Number of sessions**	**Valid trials per session**	**Interval(s)**	**Number of sessions**	**Valid trials per session**	**Interval(s)**
1	3	46/48/27	7.43 ± 4.40	3	42/34/38	7.78 ± 1.05
2	3	26/24/18	13.8 ± 4.17	3	23/19/19	14.55 ± 5.15
3	2	17/38	10.31 ± 6.05	3	32/36/28	9.42 ± 1.75
4	4	33/31/31/36	9.92 ± 4.07	3	14/38/28	9.31 ± 4.78
5	3	35/46/36	7.81 ± 2.59	2	33/34	9.37 ± 1.85
6	2	34/27	10.08 ± 6.05	3	35/22/31	10.33 ± 2.33
7	3	39/30/26	9.17 ± 2.33	3	19/19/24	15.18 ± 6.03
8	2	28/19	21.15 ± 6.88	1	27	22.48 ± 6.29

*The number of valid movements per session and the interval between the movements of all subjects*.

*Interval, Mean ± Standard Deviation (SD)*.

### S1-M1 HG power difference

All analyses were conducted using MATLAB software (MathWorks, Natick, MA, USA). ECoG data were re-referenced to a common average reference (CAR). To remove electrical device noise, we used a zero-phase-lag infinite-impulse response (IIR) notch filter (center at 60 and 120 Hz). The filtered data were epoched for a time window of 2 s before onset to 1 s after offset. To determine the HG power during active movement, we used the Morlet wavelet transform with a frequency range of 5–100 Hz (80 Hz for Subject 1), and the data were normalized by the power of each frequency of the baseline periods (−0.75 to −0.25 s). In this analysis, the effective wavelet width (95% confidence interval of the Gaussian function obtained by calculating absolute value of Morlet wavelet) at 50 Hz, which affects the temporal resolution due to the smoothing effect, was approximately 80 ms. The HG power levels of each trial were averaged across 50–100 Hz (80 Hz for Subject 1) and the movement period.

To create a brain map which represents the HG power difference between resting and movement periods, the HG power levels from each electrode were averaged across all trials. Because the scales of these levels differed among the subjects, we divided the maximum power into the power from each electrode for normalization. To test the power difference between S1 and M1 during hand grasping and elbow flexion, we chose the electrodes which showed maximum amounts of HG power in each S1 and M1 case. We also calculated the mean HG power of each S1 and M1 electrode (2–5 electrodes each). In this analysis, electrodes which were located near the central sulcus were included (1.5 cm from the central sulcus). For significance testing, we utilized the Wilcoxon signed-rank test.

### Classification

We used a movement-type classification method in order to validate the information of the HG activities from M1 and S1. For this analysis, we applied a linear support vector machine (SVM). Features were extracted by averaging the amounts of HG power during single-trial movements from the S1 and M1 electrodes. To avoid bias due to differences in the numbers of features between S1 and M1, we extracted identical numbers of features from each area. To minimize bias due to inter-session power differences, we used features from all sessions with each subject for classification. Classification performances were evaluated by 10-fold cross validation. The correct rates from each test sessions were averaged to evaluate the classification performance.

### Timing of S1 HG activity

To investigate whether or not the timing of the S1 HG power increase preceded that of the actual movement onset, we initially selected the S1 electrodes which showed the greatest HG power changes from each subject. In this analysis, the baseline period was defined as the time from –2 to –1 s of movement onset. Extracted single-trial HG and EMG power traces were normalized by the power of this baseline period. To avoid effects due to subtle changes in the EMG signals before movement onset, trials were excluded when the EMG power fluctuation before the movement onset exceeded 4 SD (standard deviation; excluded trials = 305, 7.09 trials per session). Because we could not define the exact onset time of the HG power from a single-trial trace, trials from each session were averaged based on the EMG onset time. To remove any transient bursts of HG power before movement, the power was smoothed with a window length of 100 ms. Note that this smoothing filter tended to shift the onset time-point to the pre-movement side (–x direction). After smoothing, we applied the same normalization procedure to the mean EMG and HG power traces. Finally, according to previous studies (Hotson et al., 2016; Branco et al., 2017), we defined the onset time of the averaged HG trace as the time point at which the power exceeds 2 SD. We performed this analysis again by including the 4 SD trials above.

We also applied the same procedure to the cued, 3-D center-out reaching experiment with accelerometer signals. To do this, we calculated the square sum of three-axis accelerometer signals and then marked the vertex between resting and transient period signals as the point of movement onset. S1 electrodes which showed maximum HG activities during movement were chosen. Because we focused on the HG onset time regardless of the task type, we did not categorize the reaching directions during this analysis (total: 120 trials per subject).

### Correlation between EMG and HG fluctuations before movement

To investigate the relationship between EMG and HG fluctuations before movement onset, at the outset we first calculated the SD of the pre-movement periods ranging from −0.75 to −0.1 s for the normalized data of each session. Given that the SD of the baseline period (−2 to −1 s) are 1 for both the EMG and HG signals, we could directly calculate the Pearson correlation between the SD of the pre-movement periods from the EMG and HG power levels. This analysis was repeated for the condition which included the 4 SD trials.

## Results

### HG activities in S1 are more dominant than those in M1

**Figure 1:**
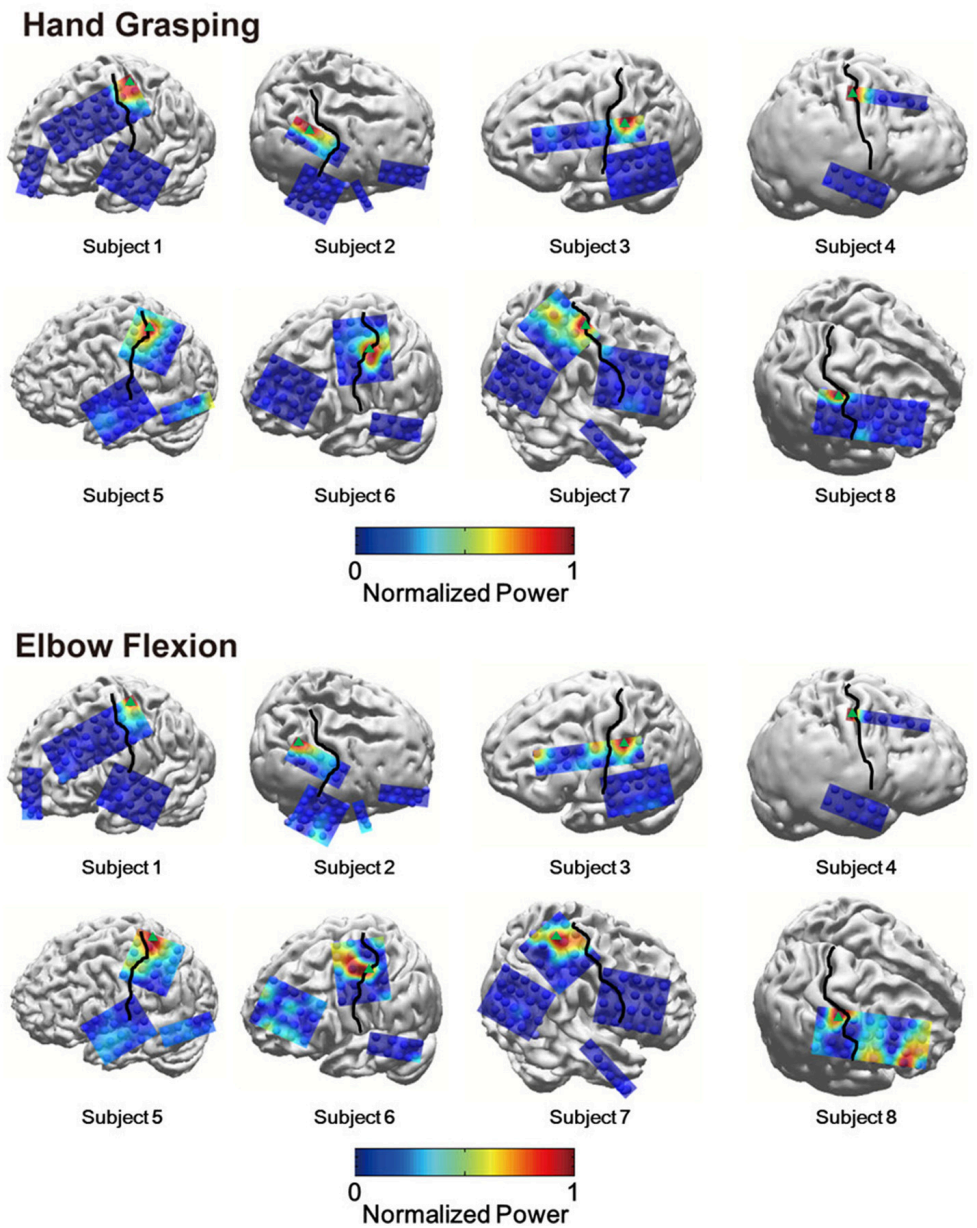
Topographical maps of spatially distributed HG power levels during hand grasping **(top)** and elbow flexion **(bottom)**. Black lines indicate the central sulcus. Green triangles indicate the electrode which showed the highest HG power among all electrodes. Some electrodes are not shown because they were located on invisible sites.

**Figure 2:**
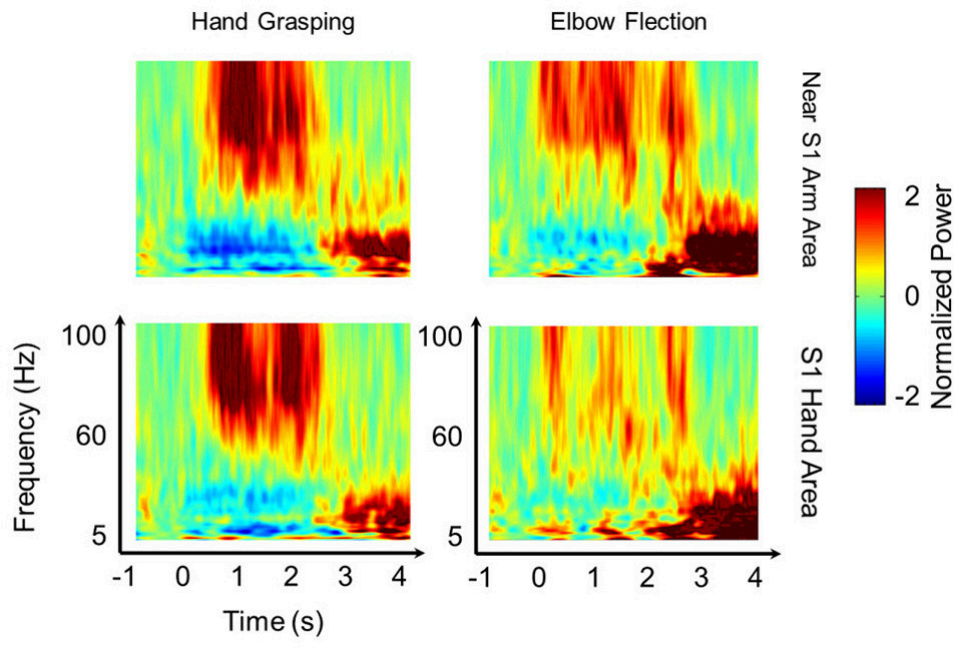
Spatio-temporal pattern of HG power. Representative time-frequency plots during hand grasping **(left)** and elbow flexion **(right)** in near the S1 arm area **(top)** and in the S1 hand area **(bottom)** from Subject 2. The results indicate that S1 HG activities show distinct power changes and show the difference not only in somatotopy but also in temporal dynamics between the two movement types. The 60 Hz power line noise was removed after frequency-by-frequency normalization.

To investigate the relative HG power difference between S1 and M1 during the voluntary hand grasping and elbow flexion trials, we initially calculated the power levels from eight subjects and mapped the relative power levels compared to the maximum power of each subject to the 3-D brain structures. Note that the 3-D brain models were constructed from individual MR images of each subject; thus, the black lines shown in Figure 1 directly indicate the central sulci of each subject. Overall, the S1 HG power changes during active movements were greater than those associated with M1 regardless of the movement type (Figure 1). The electrodes, which showed maximum HG power levels from each subject, were mainly located in the S1 area (Figure 1, green triangle). Although the power increases of M1 areas were significant in most of sessions and subjects, the maximum HG power levels in S1 were much higher than those in M1 (Supplementary Figure 2). The differences in the maximum HG power between S1 and M1 were highly significant in 14 of 16 conditions across all subjects and movement types (paired *t*-test: maximum *p* = 0.012, median *p* < 0.0001). Figure 2 shows the representative time-frequency plots from S1 hand and near elbow areas during each movement. The HG activities showed different spatio-temporal patterns depending on the movement type, consistent with the previous studies (Miller et al., 2007; Branco et al., 2017). Specifically, the temporal dynamics of HG power in S1 seemed to follow the sequence of movements (i.e., hand grasping and releasing, elbow flexion, and extension).

**Figure 3:**
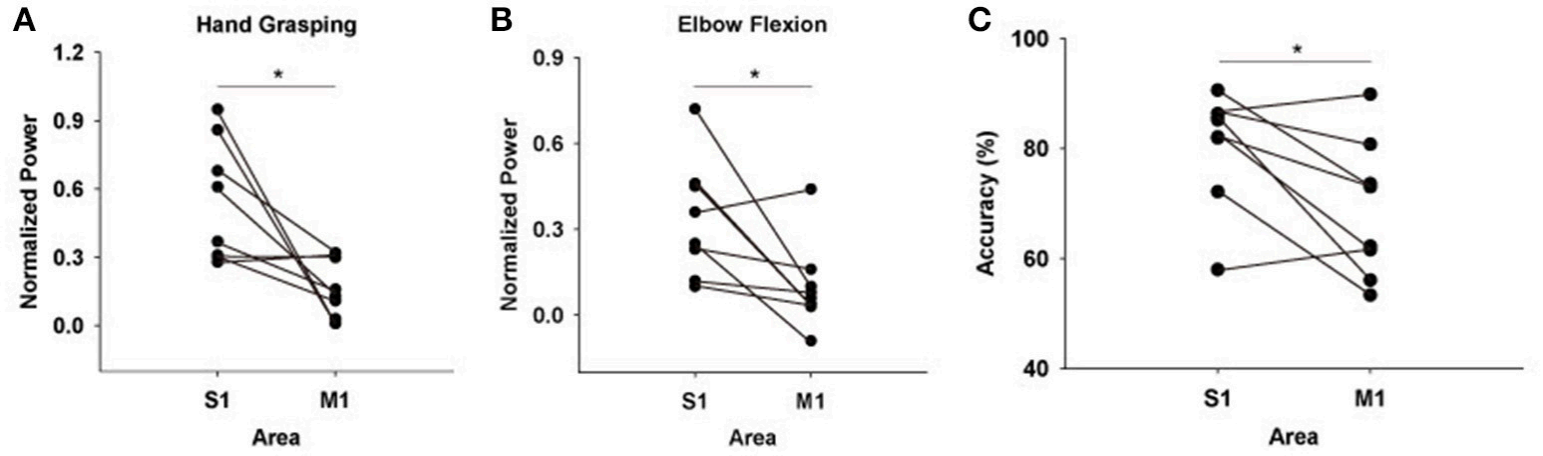
Mean HG power differences between S1 and M1 electrodes across all subjects during **(A)** hand grasping and **(B)** elbow flexion [mean power values across all subjects: S1 hand grasping = 0.54 ± 0.095 (mean ± standard error), M1 hand grasping = 0.17 ± 0.044, S1 elbow flexion = 0.34 ± 0.073, M1 elbow flexion = 0.011 ± 0.054]. Power values of all sessions from each subject were averaged. The y-axis (normalized power) uses an arbitrary unit scale. **(C)** Differences in classification accuracies between S1 and M1 features across all subjects. Black dots indicate the respective power or accuracy level of each subject. ^*^p < 0.05 (Wilcoxon signed-rank test)

Although the results were quite consistent among subjects, it is possible for the areas which showed maximum amounts of HG power to vary depending on the placement of ECoG electrode grid. Thus, we also calculated overall HG power levels from each S1 and M1 electrodes. Among all subjects, the mean values for the HG power from the S1 electrodes were greater than those from M1 electrodes both in hand grasping and elbow flexion conditions, except for one subject (Figures 3A,B).

Next, we tested how much the HG activities from S1 provide informative signals for movement type classification compared to those from M1. To do this, we extracted features from the single-trial HG power levels of each area, after which calculated classification accuracy in each case with a linear SVM. Interestingly, the classification accuracy from the M1 features was significantly lower than that from the S1 features (Figure 3C; Wilcoxon signed-rank test: *n* = 8, *p* < 0.05). The mean classification accuracy values over S1 and M1 across all subjects were 80.22 ± 3.69% (mean ± standard error) and 68.79 ± 4.48%, respectively.

### S1 HG activity mainly represents somatosensory feedback

**Figure 4:**
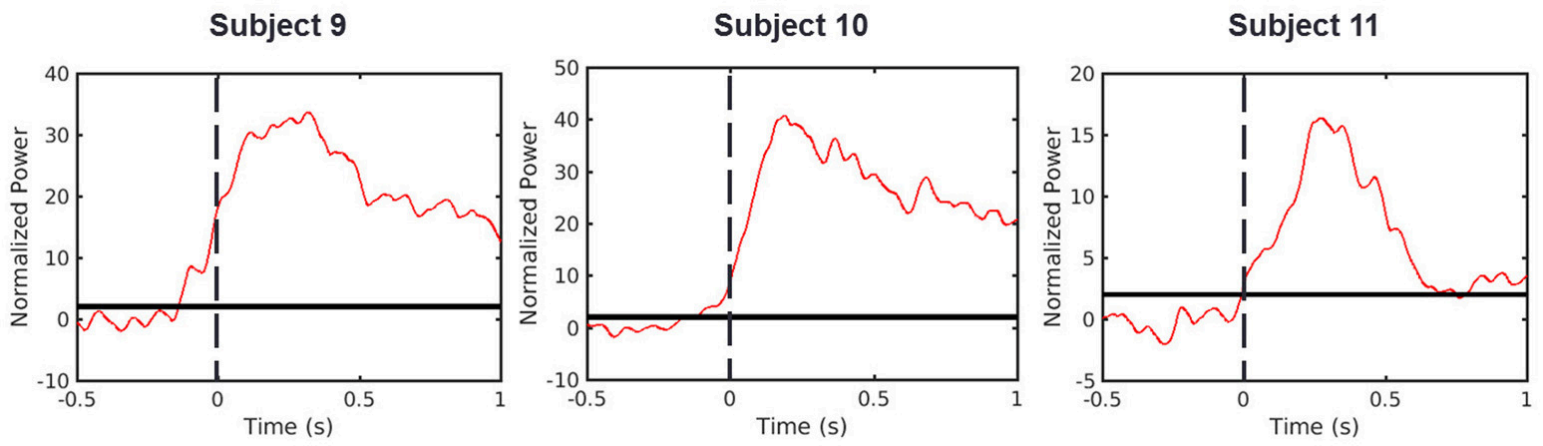
HG power time traces during cued movements. Red lines denote averaged HG time traces. Dashed line represents the movement onset as determined by the accelerometer signal. Solid black lines indicate the chance levels (2 SD) of the HG power increases. Calculated onset time points from all subjects were lower than zero (Subject 9 = −0.14 s, Subject 10 = −0.13 s, Subject 11 = −0.01 s).

Recent findings have indicated that there is a significant S1 HG power increase before movement onset representing neural activation beyond somatosensory feedback (Sun et al., 2015; Hotson et al., 2016; Branco et al., 2017). However, determining the mechanism reasonable for the HG activity in S1 prior to movement onset remains challenging. To address this, we initially replicated the result of these previous studies. We evaluated the timing of HG activity from our dataset, which was recorded in the cued, 3-D center-out arm movement paradigm. In this experiment, the onset time of each movement was determined by three-axis accelerometer which was attached to the index finger of each subject. Indeed, we obtained similar results from S1 electrodes of all three subjects (Figure 4). In these electrodes, the HG activities became significant 140, 130, and 10 ms before movement onset in Subjects 9, 10, and 11, respectively. However, although external location sensors such as, accelerometer can define the movement onset time, it is difficult to detect subtle muscle contractions prior to the movement, which may induce undesirable HG power changes. Moreover, the visual cue to initiate movement can affect the HG power as a stimulus unrelated to the movement preparation. Therefore, in order to evaluate the relationship between the brain and muscle activities more reliably, the results from previous studies and our analysis needed to be confirmed using EMG signals during fully voluntary movements.

**Figure 5:**
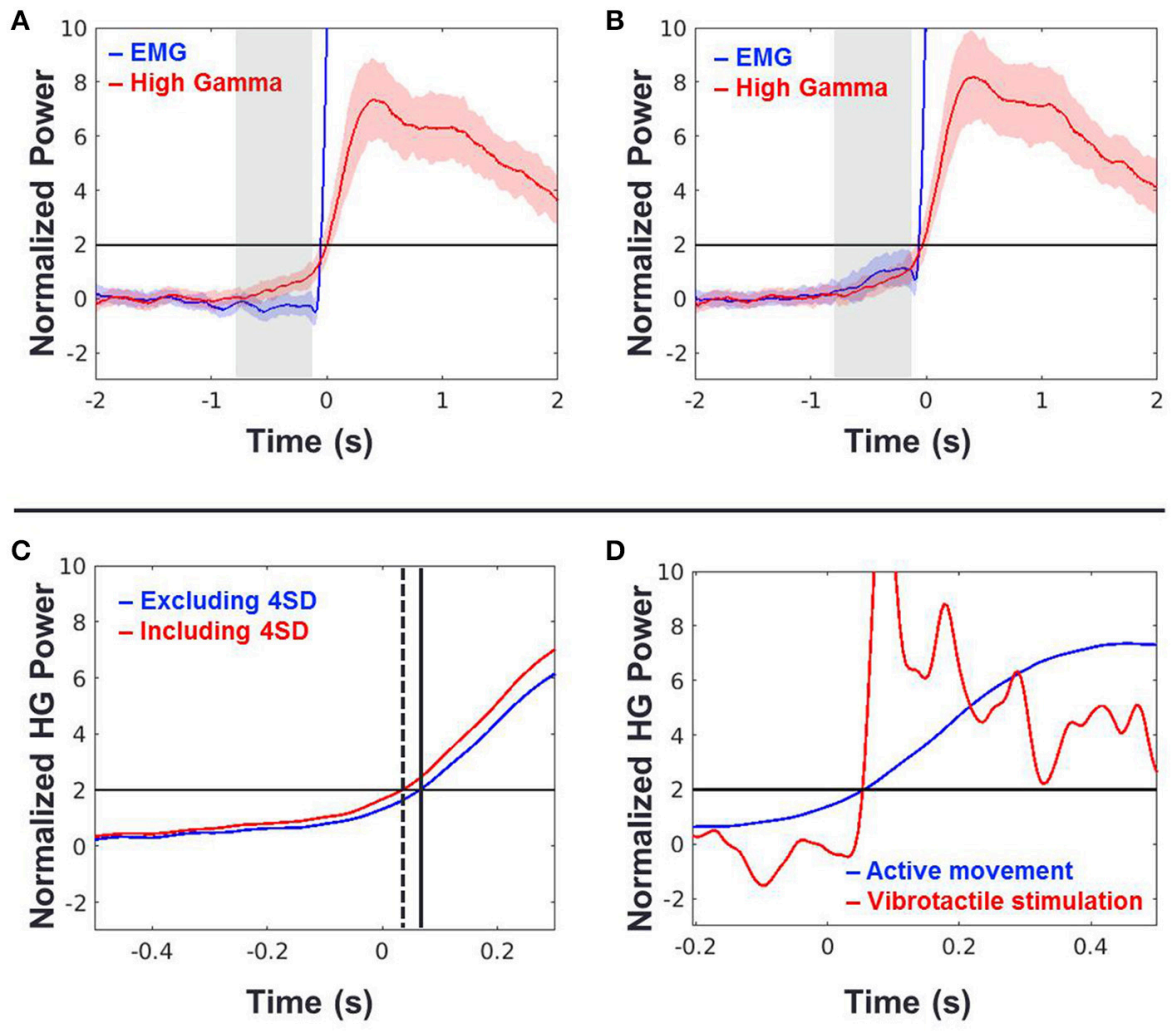
**(A,B)** Time traces of grand-averaged EMG (blue) and HG power (red) across all movement types, sessions and subjects when the 4 SD trials were excluded **(A)** and included **(B)**. Black horizontal lines indicate the chance levels (2 *SD*) of the EMG and HG power increases. Shaded areas with the color corresponding to the respective conditions denote 95% confidence intervals. Shaded areas shown in gray indicate periods of −0.75 to −0.1 s of the movement onset used in the further analysis. **(C)** Time shift of HG onset between (**A**; blue) and (**B**; red) conditions. Time *t* = 0 indicates the movement onset time-point determined by the EMG signal. Dashed and solid vertical lines indicate the HG onset time-points of **(A,B)** conditions, respectively. **(D)** HG power time traces of (**A**; blue) and vibrotactile stimulation (red). Time *t* = 0 indicates the movement onset or vibrotactile stimulus onset. Note that the HG onset timings of these two conditions are virtually the same.

To do this, we calculated the onset timing of HG activities from 43 sessions of 8 subjects during voluntary hand grasping and elbow flexion using EMG onset time points. In this analysis, trials were excluded if the pre-movement EMG signals exceed 4 SD compared to the baseline periods (−2 to −1 s of movement onset). The averaged HG onset time across all sessions and subjects was 49 ± 25 ms (mean ± standard error) after movement onset. The HG onset occurred significantly later than movement onset time (one-tailed *t*-test, *n* = 43, *p* < 0.027; Figure 5A). We also performed the same analysis while including the rejected trials above (Figure 5B). The HG time trace was slightly shifted to the left as compared to when these trials were excluded (Figure 5C), but the mean onset time was still later than movement onset (31 ms) although the difference was not significant. Next, we compared the HG onset time from voluntary movement to that from passive vibrotactile stimulation (Figure 5D). There was no difference between these two conditions (two-sample *t*-test, *n* = 93, *p* = 0.92).

**Figure 6:**
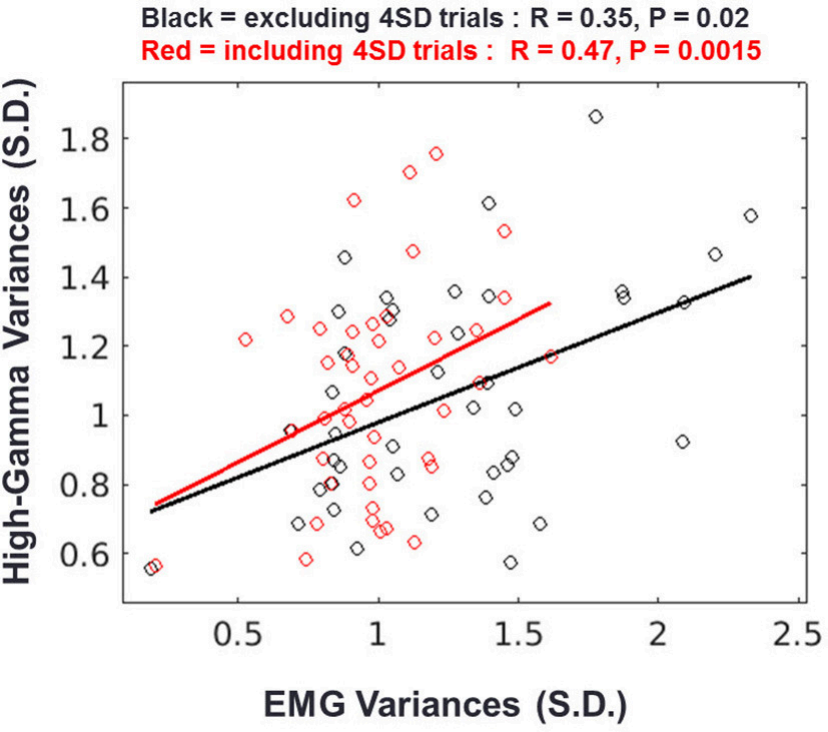
Relationship between HG and EMG fluctuations during the pre-movement period. Result of regression analysis when the 4 *SD* trials are excluded (black) or included (red). Each circle represents the standard deviations of the HG power and EMG signal from one session. Solid lines indicate the regression slopes from the two conditions.

Although we showed that the HG onset occurs after movement, the calculated onset time-point of HG can change according to the detection criteria. Furthermore, there exists a slow increase in power before movement, although its power level was not statistically significant (see the shaded area in Figure 5A). Therefore, we investigated the relationship between the pre-movement EMG signal and HG power fluctuations to confirm that these activities are also related to the motor-evoked somatosensory information. To do this, we normalized these two types of data from each session using signals from the baseline period (−2 to −1 s of movement onset) and then calculated the Pearson correlation between the SD of HG power and the EMG fluctuation during the pre-movement period (−0.75 to −0.1 s). Interestingly, we found a significant correlation between them (Figure 5, black; *n* = 43, *r* = 0.35, *p* = 0.02). Furthermore, the relationship between them became stronger with including the aforementioned 4 *SD* trials (Figure 6, red; *r* = 0.47, *p* = 0.0015). These results indicate that a subtle EMG power fluctuation can affect the increase in the HG power even during pre-movement period.

## Discussion

In this study, we found that HG activities in the primary somatosensory cortex (S1) are more dominant than those in primary motor cortex (M1) during active, voluntary movement. Our classification results indicate that the movement-related HG signals in S1 are more informative than those in M1. Through these findings, we suggest that movement-related S1 activities strongly affect the overall sensorimotor HG power. Although recent ECoG studies have indicated that HG signals from S1 provide feature as good as those from M1 (Pistohl et al., 2012; Branco et al., 2017), no investigations exist to quantify the influence of HG activity in S1 during voluntary movement from a large number of datasets (43 sessions from 8 subjects). Although our results have several limitations with regard to generalizing this phenomenon due to the placement and spatial resolution of standard ECoG grid and the number of types of movements, the important aspect here is that neuronal somatosensory feedback during active movement may play more crucial roles than originally expected.

In the present study, we also found that the onset time of movement-related somatosensory HG power follows that of actual movement and that the small HG fluctuation of the pre-movement period is closely related to subtle muscle contractions during that period. This indicates that even if a small amount of S1 HG changes is detected during the pre-movement period, these activities hardly represent neural processing for the movement preparation. Rather, they possibly reflect somatosensory feedback for the subtle muscle activity or the efferent modulation of sensory input by S1. Previous studies have investigated the onset timing of S1 HG activity by using motion or pressure sensors, and suggested that significant S1 HG activity before the determined movement onset represents the neural interaction for movement preparation (Sun et al., 2015; Hotson et al., 2016; Branco et al., 2017). However, the sensors could scarcely detect subtle or isometric muscle contractions before a desired movement task was performed. With EMG for movement onset detection, we obtained contradictory results with a relatively large number of both sessions and subjects. Therefore, given our results and the additional reasons given below, we suggest that somatosensory HG activities during active movement mainly represent cortical neural processing for movement-related somatosensory feedback. First, the power changes of HG responses in S1 during passive somatosensory stimulation are as robust as those during active movement tasks (Avanzini et al., 2016; Hotson et al., 2016). This trend was also confirmed in our present result with vibrotactile stimulation. Although the HG activity is modulated by attention and other neuronal interaction (Bauer et al., 2006; Canolty et al., 2006; Ray et al., 2008c), it is clear that this activity robustly represents the properties of sensory stimulation given its intensity dependence and stimulus-quality-specific activation pattern (Womelsdorf et al., 2006; Ray et al., 2008b; Rossiter et al., 2013). Second, the absolute power value of the HG activity in S1 during an attempted movement without somatosensory feedback is substantially lower than that during active movement or tactile stimulation (Yanagisawa et al., 2012; Wang et al., 2013). If sensorimotor interaction such as, efference copy mainly contributes to the overall somatosensory HG activity, a drastic change in the HG power in S1 should be found in patients with tetraplegia because their internal circuitry of their brain is still functional despite the fact its efficiency may be lower than that of intact people. Third, based on our onset timing result, it is unlikely that the HG signal in S1 before movement represents neural activity for movement preparation. Therefore, a prominent HG activation in S1 during movement is mainly induced by somatosensory information from the periphery.

In this study, we found a significant correlation between the HG fluctuation in S1 and the subtle muscle activation during pre-movement period, suggesting that this fluctuation may not be related to the sensorimotor interaction for movement preparation. However, this does not mean that the activity solely represents the somatosensory feedback itself. Specifically, it is possible that this S1 activity might be partially related to other sensory mechanisms, such as, the corticospinal efferent modulation of sensory input by S1, although the relationship between the HG and efferent modulation by S1 has not been elucidated yet (Lemon, 2008).

Although our results imply the possibility of considerable performance inflation in sensorimotor HG-based BMI due to somatosensory feedback, it is unlikely that the HG signals in S1 without somatosensory feedback would provide uninformative features for BMI systems. As noted above, studies of patient with tetraplegia have reported a weak but distinct HG pattern in both the sensory and motor areas during attempted movement, although the decoding performance using this signal was relatively low (Yanagisawa et al., 2012; Hochman et al., 2013). Furthermore, there is evidence that the somatosensory cortex receives information from the premotor cortex during voluntary movement without somatosensory feedback (Christensen et al., 2007). Nevertheless, however, it remains inconclusive as to whether the HG activity in S1 also represents neural activity pertaining to sensorimotor interaction, such as, an efference copy. Thus, further investigations are required to reveal the mechanism of neuronal HG activity during sensorimotor interaction in the event of movement.

A recent intrasulcal ECoG study has indicated that there also exists robust HG activation in the pre- and post-central sulcus during attempted movements (Morris et al., 2015). Although we showed that the mean HG power levels across the post-central gyrus are greater than those across the pre-central gyrus, it is possible that the HG patterns in the sulcus might be quite different because of the very focal nature of the HG activity (Yanagisawa et al., 2009; Branco et al., 2017). Hence, it will be of interest to confirm whether these patterns can be conserved when the activities from the pre- and post-central sulci are included.

In summary, we for the first time statistically quantified the influence of HG activity in S1 during voluntary movement. We found prominent S1 HG activity which is more dominant and informative than that in M1 during voluntary movement, suggesting that this activity strongly affects the overall sensorimotor HG power. We also suggest that the onset time of HG activity in S1 is significantly later than the movement onset time, and even a small amount of HG fluctuation prior to movement is closely related to the subtle muscle activation occurring during that period. These results imply that HG activation in S1 during voluntary movement is mainly induced by the somatosensory information from the periphery and not by sensorimotor interactions to prepare for movement.

## Ethics statement

This study was carried out in accordance with the recommendations of “Institutional Review Board of Seoul National University Hospital (H-0912-067-304)” with written informed consent from all subjects. All subjects gave written informed consent in accordance with the Declaration of Helsinki. The protocol was approved by the “Institutional Review Board of Seoul National University Hospital.”

## Author contributions

JK and CC designed the research. CC performed the surgeries. SR and EJ performed the experiment. SR, EJ, and JK analyzed the data. SR, JK, and CC wrote the paper.

### Conflict of interest statement

The authors declare that the research was conducted in the absence of any commercial or financial relationships that could be construed as a potential conflict of interest.

## References

[B1] Adams R. A. Shipp S. Friston K. J. (2013). Predictions not commands: active inference in the motor system. Brain Struct. Funct. 218, 611–643. 10.1007/s00429-012-0475-5 23129312 PMC3637647

[B2] Armand J. Oliver E. Edgley S. A. Lemon R. N. (1997). The postnatal development of corticospinal projections from motor cortex to the cervical enlargement in the macaque monkey. J. Neurosci. 17, 251–266. 8987753 10.1523/JNEUROSCI.17-01-00251.1997 PMC6793701

[B3] Avanzini P. Abdollahi R. O. Sartori I. Caruana F. Pelliccia V. Casaceli G.. (2016). Four-dimensional maps of the human somatosensory system. Proc. Natl. Acad. Sci. U.S.A. 113, E1936–E1943. 10.1073/pnas.1601889113 26976579 PMC4822602

[B4] Bauer M. Oostenveld R. Peeters M. Fries P. (2006). Tactile spatial attention enhances gamma-band activity in somatosensory cortex and reduces low-frequency activity in parieto-occipital areas. J. Neurosci. 26, 490–501. 10.1523/JNEUROSCI.5228-04.2006 16407546 PMC6674422

[B5] Bleichner M. G. Freudenburg Z. V. Jansma J. M. Aarnoutse E. J. Vansteensel M. J. Ramsey N. F. (2016). Give me a sign: decoding four complex hand gestures based on high-density ECoG. Brain Struct. Funct. 221, 203–216. 10.1007/s00429-014-0902-x 25273279 PMC4720726

[B6] Branco M. P. Freudenburg Z. V. Aarnoutse E. J. Bleichner M. G. Vansteensel M. J. Ramsey N. F. (2017). Decoding hand gestures from primary somatosensory cortex using high-density ECoG. Neuroimage 147, 130–142. 10.1016/j.neuroimage.2016.12.004 27926827 PMC5322832

[B7] Canolty R. T. Edwards E. Dalal S. S. Soltani M. Nagarajan S. S. Kirsch H. E.. (2006). High gamma power is phase-locked to theta oscillations in human neocortex. Science 313, 1626–1628. 10.1126/science.1128115 16973878 PMC2628289

[B8] Chestek C. A. Gilja V. Blabe C. H. Foster B. L. Shenoy K. V. Parvizi J.. (2013). Hand posture classification using electrocorticography signals in the gamma band over human sensorimotor brain areas. J. Neural Eng. 10:026002. 10.1088/1741-2560/10/2/026002 23369953 PMC3670711

[B9] Cheyne D. Bells S. Ferrari P. Gaetz W. Bostan A. C. (2008). Self-paced movements induce high-frequency gamma oscillations in primary motor cortex. Neuroimage 42, 332–342. 10.1016/j.neuroimage.2008.04.178 18511304

[B10] Christensen M. S. Lundbye-Jensen J. Geertsen S. S. Petersen T. H. Paulson O. B. Nielsen J. B. (2007). Premotor cortex modulates somatosensory cortex during voluntary movements without proprioceptive feedback. Nat. Neurosci. 10, 417–419. 10.1038/nn1873 17369825

[B11] Crone N. E. Miglioretti D. L. Gordon B. Lesser R. P. (1998). Functional mapping of human sensorimotor cortex with electrocorticographic spectral analysis - II. Event-related synchronization in the gamma band. Brain 121, 2301–2315. 10.1093/brain/121.12.2301 9874481

[B12] Hochman S. Wang W. Collinger J. L. Degenhart A. D. Tyler-Kabara E. C. Schwartz A. B. (2013). An electrocorticographic brain interface in an individual with tetraplegia. PLoS ONE 8:e55344 10.1371/journal.pone.0055344 23405137 PMC3566209

[B13] Hotson G. McMullen D. P. Fifer M. S. Johannes M. S. Katyal K. D. Para M. P. (2016). Individual finger control of a modular prosthetic limb using high-density electrocorticography in a human subject. J. Neural Eng. 13:026017 10.1088/1741-2560/13/2/026017 26863276 PMC4875758

[B14] Jenmalm P. Johansson R. S. (1997). Visual and somatosensory information about object shape control manipulative fingertip forces. J. Neurosci. 17, 4486–4499. 9151765 10.1523/JNEUROSCI.17-11-04486.1997 PMC6573538

[B15] Lemon R. N. (2008). Descending pathways in motor control. Annu. Rev. Neurosci. 31, 195–218. 10.1146/annurev.neuro.31.060407.125547 18558853

[B16] Lim M. Kim J. S. Chung C. K. (2012). Modulation of somatosensory evoked magnetic fields by intensity of interfering stimuli in human somatosensory cortex: an MEG study. Neuroimage 61, 660–669. 10.1016/j.neuroimage.2012.04.003 22516368

[B17] Miller K. J. Leuthardt E. C. Schalk G. Rao R. P. N. Anderson N. R. Moran D. W. (2007). Spectral changes in cortical surface potentials during motor movement. J. Neurosci. 27, 2424–2432. 10.1523/JNEUROSCI.3886-06.2007 17329441 PMC6673496

[B18] Morris S. Hirata M. Sugata H. Goto T. Matsushita K. Yanagisawa T.. (2015). Patient-specific cortical electrodes for sulcal and gyral implantation. IEEE Trans. Biomed. Eng. 62, 1034–1041. 10.1109/TBME.2014.2329812 25029330

[B19] Pistohl T. Schulze-Bonhage A. Aertsen A. Mehring C. Ball T. (2012). Decoding natural grasp types from human ECoG. Neuroimage 59, 248–260. 10.1016/j.neuroimage.2011.06.084 21763434

[B20] Ray S. Crone N. E. Niebur E. Franaszczuk P. J. Hsiao S. S. (2008a). Neural correlates of high-gamma oscillations (60–200 Hz) in macaque local field potentials and their potential implications in electrocorticography. J. Neurosci. 28, 11526–11536. 10.1523/JNEUROSCI.2848-08.2008 18987189 PMC2715840

[B21] Ray S. Hsiao S. S. Crone N. E. Franaszczuk P. J. Niebur E. (2008b). Effect of stimulus intensity on the spike-local field potential relationship in the secondary somatosensory cortex. J. Neurosci. 28, 7334–7343. 10.1523/JNEUROSCI.1588-08.2008 18632937 PMC2597587

[B22] Ray S. Niebur E. Hsiao S. S. Sinai A. Crone N. E. (2008c). High-frequency gamma activity (80–150 Hz) is increased in human cortex during selective attention. Clin. Neurophysiol. 119, 116–133. 10.1016/j.clinph.2007.09.136 18037343 PMC2444052

[B23] Rossiter H. E. Worthen S. F. Witton C. Hall S. D. Furlong P. L. (2013). Gamma oscillatory amplitude encodes stimulus intensity in primary somatosensory cortex. Front. Hum. Neurosci. 7:362. 10.3389/fnhum.2013.00362 23874282 PMC3711008

[B24] Rothwell J. C. Traub M. M. Day B. L. Obeso J. A. Thomas P. K. Marsden C. D. (1982). Manual motor performance in a deafferented man.pdf. Brain 105, 515–542. 10.1093/brain/105.3.515 6286035

[B25] Ryun S. Kim J. S. Lee S. H. Jeong S. Kim S.-P. Chung C. K. (2014). Movement type prediction before its onset using signals from prefrontal area: an electrocorticography study. Biomed Res. Int. 2014, 1–9. 10.1155/2014/783203 25126578 PMC4122137

[B26] Sanes J. N. Mauritz K.-H. Evarts E. V. Dalakas M. C. Chu A. (1984). Motor deficits in patients with large-fiber sensory neuropathy. Proc. Natl. Acad. Sci. U.S.A. 81, 979–982. 10.1073/pnas.81.3.979 6322181 PMC344963

[B27] Sun H. Blakely T. M. Darvas F. Wander J. D. Johnson L. A. Su D. K.. (2015). Sequential activation of premotor, primary somatosensory and primary motor areas in humans during cued finger movements. Clin. Neurophysiol. 126, 2150–2161. 10.1016/j.clinph.2015.01.005 25680948 PMC4512926

[B28] Wang W. Collinger J. L. Degenhart A. D. Tyler-Kabara E. C. Schwartz A. B. Moran D. W.. (2013). An electrocorticographic brain interface in an individual with tetraplegia. PLoS ONE 8:e55344. 10.1371/journal.pone.0055344 23405137 PMC3566209

[B29] Wiest M. C. Nicolelis M. A. (2003). Behavioral detection of tactile stimuli during 7–12 Hz cortical oscillations in awake rats. Nat. Neurosci. 6, 913–914. 10.1038/nn1107 12897789

[B30] Womelsdorf T. Fries P. Mitra P. P. Desimone R. (2006). Gamma-band synchronization in visual cortex predicts speed of change detection. Nature 439, 733–736. 10.1038/nature04258 16372022

[B31] Yanagisawa T. Hirata M. Saitoh Y. Kato A. Shibuya D. Kamitani Y. (2009). Neural decoding using gyral and intrasulcal electrocorticograms. Neuroimage 45, 1099–1106. 10.1016/j.neuroimage.2008.12.069 19349227

[B32] Yanagisawa T. Hirata M. Saitoh Y. Kishima H. Matsushita K. Goto T. (2012). Electrocorticographic control of a prosthetic arm in paralyzed patients. Ann. Neurol. 71, 353–361. 10.1002/ana.22613 22052728

[B33] Yeom H. G. Kim J. S. Chung C. K. (2013). Estimation of the velocity and trajectory of three-dimensional reaching movements from non-invasive magnetoencephalography signals. J. Neural Eng. 10:026006. 10.1088/1741-2560/10/2/026006 23428826

[B34] Zhang Z. G. Hu L. Hung Y. S. Mouraux A. Iannetti G. D. (2012). Gamma-band oscillations in the primary somatosensory cortex–a direct and obligatory correlate of subjective pain intensity. J. Neurosci. 32, 7429–7438. 10.1523/JNEUROSCI.5877-11.2012 22649223 PMC6703598

