# Supplementary figures and images for: Movement-Related Sensorimotor High-Gamma Activity Mainly Represents Somatosensory Feedback
Source: Front Neurosci. 2017 Jul 14;11:408. doi: 10.3389/fnins.2017.00408 (PMC5509940; doi:10.3389/fnins.2017.00408)

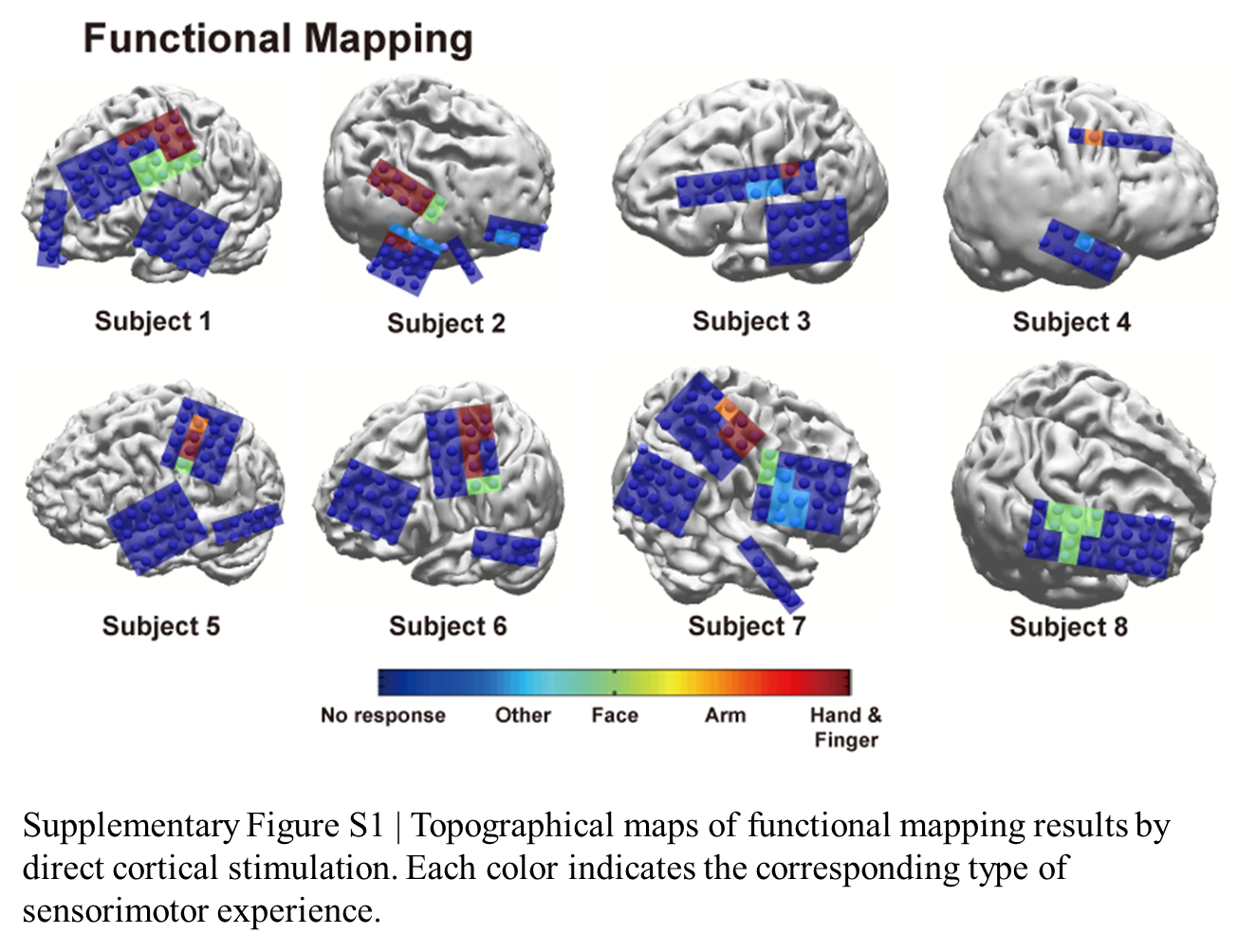

Supplement: Supplementary file 1 [file Image1.png]

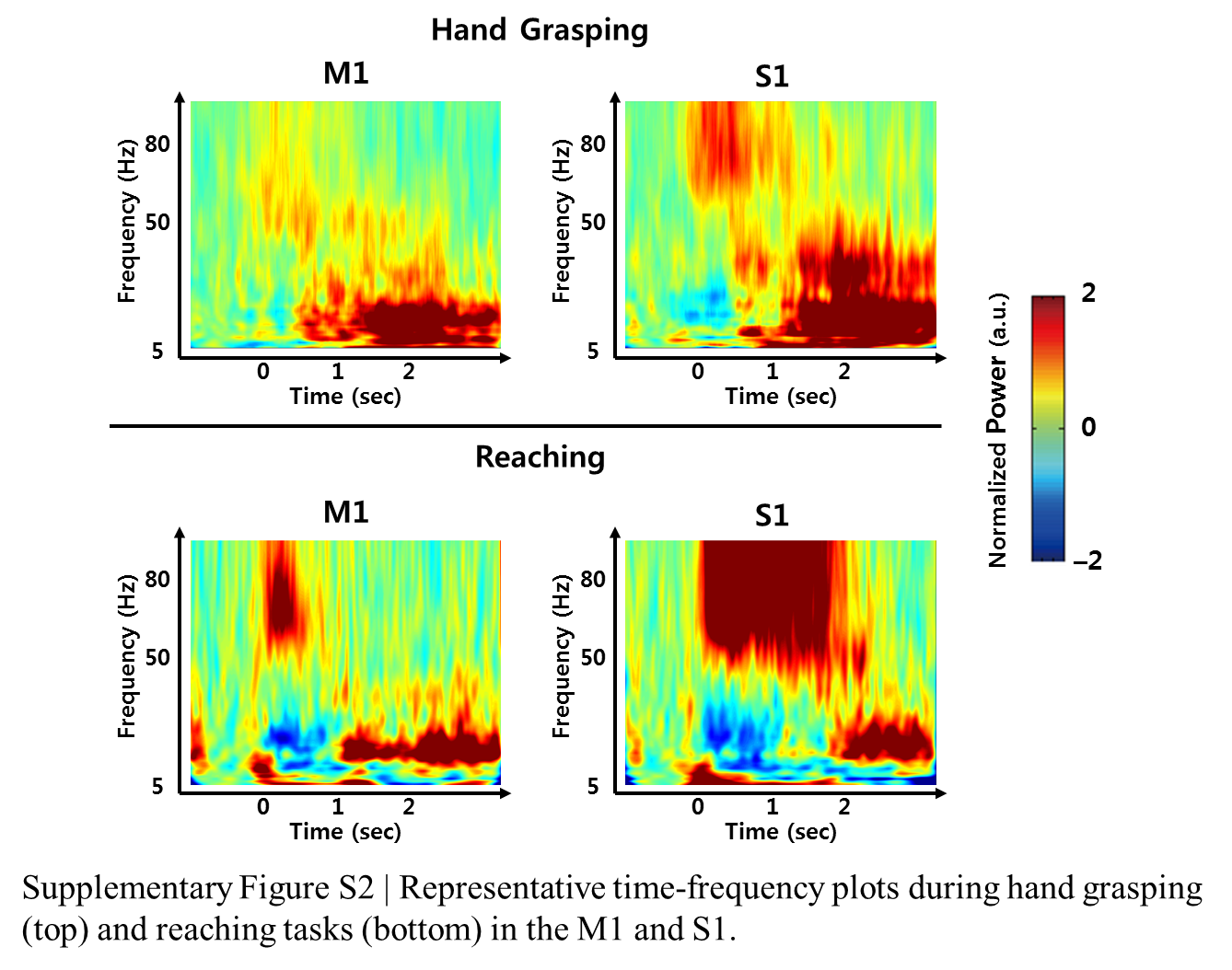

Supplement: Supplementary file 2 [file Image2.png]
